# Post-fire environmental assessment: a participatory multi-criteria approach for estimating soil erosion risk and vegetation recovery potential

**DOI:** 10.1016/j.mex.2025.103647

**Published:** 2025-09-23

**Authors:** Irina Cristal, Pere Pons, Marina Palmero-Iniesta, Elena Puigdemasa

**Affiliations:** a Animal Biology Lab and BioLand, Department of Environmental Sciences, University of Girona, Girona, Spain; b Forest Science and Technology Center of Catalonia, Solsona, Spain; c Department of Agriculture, Livestock, Fisheries and Food, Generalitat de Catalunya, Barcelona, Spain

**Keywords:** Wildfire impact assessment, MCA, Analytical hierarchy process, AHP, Soil erosion susceptibility, Vegetation regeneration

## Abstract

Although often perceived as destructive, wildfires play a vital ecological role in maintaining the function and biodiversity of fire-adapted ecosystems. However, increasing frequency, intensity, and altered seasonality of fires impede ecosystem recovery by depleting seed banks and disrupting plant maturation cycles. Post-fire soil erosion, usually triggered by the first rains after a fire event, exacerbates these challenges by accelerating nutrient loss and hindering vegetation regeneration, which can lead to ecosystem degradation. To address this risk, it is important to understand the ecosystems’ responses to fire. Here, we outline a post-fire ecosystem assessment framework that applies geo-spatial data and participatory multi-criteria analysis (MCA) based on expert judgement and local knowledge. Our methodology includes:

• Identifying key factors influencing post-fire soil erosion and vegetation recovery
• Assessing and mapping postfire soil erosion risk and vegetation recovery potential
• Validating the framework with field data and checking the stability of MCA parameters through sensitivity analysis.

This paper extends the original research article by Cristal et al. (2025), offering a comprehensive guide for researchers and environmental professionals on applying participatory MCA in post-fire environmental impact assessments.

## Graphical abstract

**Figure:**
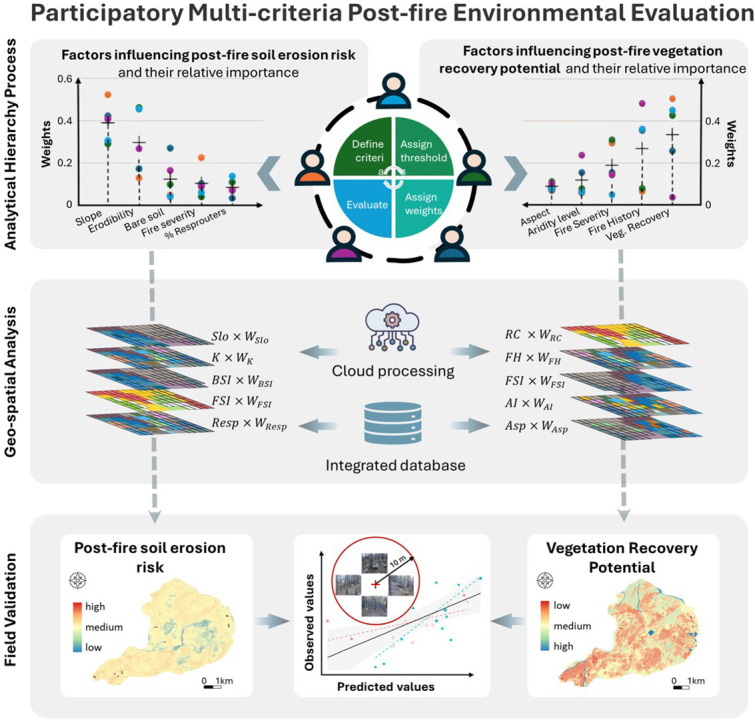

**Specifications table**

**Table:** 

**Subject area**	Environmental Science
**More specific subject area**	Post-fire environmental impact assessment
**Name of your method**	Participatory multi-criteria wildfire impact assessment
**Name and reference of original method**	Analytical Hierarchy Process (AHP, Saaty 197, 1980), Simple Additive Weighting Method (SAWM [1],)
**Resource availability**	*MCA code* DOI: 10.5281/zenodo.15119754 *Validation dataset* DOI: 10.5281/zenodo.15168015 *Validation code* DOI: https://doi.org/10.5281/zenodo.15168292 *Code repository*: https://github.com/iri-cri/MCA_Postfire_GEE

## Background

Wildfires play an important role in ecosystem dynamics by promoting fire-adapted species regeneration, nutrient cycling, and habitat heterogeneity [2]. However, shifting fire regimes driven by climate change, changes in land use, and fire management legacies, have increased the frequency, intensity, and unpredictability of wildfires, posing challenges to ecosystem recovery [3]. Among the pressing concerns are the post-fire soil erosion risk and the potential of vegetation to recover [4]. The loss of stabilizing vegetation and organic matter, combined with rainfall events shortly after fire, can trigger severe soil erosion, depleting nutrients and hindering ecosystem recovery [5]. On the other hand, increased frequency and severity of wildfires can compromise the inherent regeneration mechanisms of fire-adapted plant species. Consequently, there is a growing need for robust, evidence-based assessment tools to evaluate post-fire environmental vulnerability and guide management decisions. Multi-Criteria Analysis (MCA) emerged as a decision-support framework in environmental management, particularly valuable in situations requiring integration of diverse perspectives to ensure democratic decision-making. Nevertheless, MCA has been widely used in environmental assessments (cf [6].) and incorporated into spatial analyses (cf [7].). Environmental multi-criteria analysis typically seeks to assess environmental impacts based on predefined indicators, with the underlying assumption that each indicator contributes differently to the measured environmental characteristic. These impacts may vary in magnitude and can follow either linear or non-linear relationships, making MCA particularly useful for modelling complex ecological interactions where multiple factors interplay.

Several studies (e.g. [8,9]) have applied MCA to assess post-fire environmental impacts; however, this approach remains relatively uncommon. Outside the MCA framework [10],) estimated post-fire ecosystem vulnerability, while Underwood et al. [11] prioritized restoration areas based on ecosystem recovery potential. These studies rely on geospatial data and biophysical indicators, incorporating expert judgment, yet they do not explicitly adopt a participatory approach. While environmental assessments are primarily analytical, integrating participatory elements enhances the evaluation process by considering local knowledge alongside expert insights from multiple disciplines.

Our methodology article expands on Cristal et al. and builds upon the theoretical framework of Alloza et al. [12]. It presents a structured spatial multi-criteria analysis of factors influencing post-fire soil erosion and vegetation recovery potential. The study details the selection and derivation of key environmental factors and employs a participatory analytical hierarchy process (AHP) to integrate local and expert knowledge in weighing these factors. Sensitivity analysis based on Monte Carlo simulations, assess the robustness of the weigh3ing schemes. The framework is validated through field assessments in two wildfire-affected areas in central Spain. By ensuring transparency and reproducibility, this article provides a valuable tool for researchers and practitioners in post-fire environmental assessment.

## Method details

### Multi-criteria analysis

Multi-criteria analysis or evaluation is an umbrella term for an array of tools aimed to analyze different criteria to evaluate an outcome. One of these tools is the Analytical Hierarchy Process [13,14] that decomposes complex problems into a hierarchy of criteria. It relies on pairwise comparisons to quantify the relative importance of each criterion using a numerical scale (Supplementary Table 1). The comparisons are organized in a square matrix of *n* criteria:

$$A=\left[\begin{array}{ccccc} 1 & a_{12} & a_{13} & \ldots & a_{1n}\\ \frac{1}{a_{12}} & 1 & a_{23} & \ldots & a_{2n}\\ \frac{1}{a_{13}} & \frac{1}{a_{23}} & 1 & \ldots & a_{3n}\\ \vdots & \vdots & \vdots & \ddots & \vdots \\ \frac{1}{a_{1n}} & \frac{1}{a_{2n}} & \frac{1}{a_{3n}} & \ldots & 1 \end{array}\right]$$

*Where: the criteria are arranged along both the rows and columns; each element a_ij_ represents the relative importance of criterion* i *over criterion* j*; the diagonal elements are always 1, since a criterion compared to itself has equal importance; the matrix is reciprocal, meaning a_ji_ = 1/a_ij_.*

The matrix is then normalized by dividing each element by the sum of each column: $N_{ij}=\frac{a_{ij}}{\sum_{i=1}^{n}a_{ij}}$ .

Finally, the weights for each criterion are obtained by averaging each row: $w_{i}=\frac{1}{n}\sum_{j=1}^{n}N_{ij}$, resulting in a weight vector: $W={\left[w_{1},\;w_{2},\;w_{3},\;\ldots \;w_{n}\right]}^{T}$

An important step in AHP is checking the consistency ratio (CR) to ensure that the judgments are consistent, with a CR of <0.1. CR is calculated as follows:

$$CR=\;CI\;/\;RI,\;\text{where}\;CI=\frac{\lambda_{max}\;-\;n}{n\;-\;1}$$

*where CI the consistency index and RI the random index based on the number of criteria (Supplementary Table 2), λ_max_ is the maximum eigenvalue of the pairwise comparison matrix and n is the number of criteria.*

The final ranking is obtained by multiplying the criteria weights with the scores each criterion: $=W^{T}X$, where $W^{T}$ is the weight matrix and X is the criteria score matrix.

AHP is widely used in environmental evaluations, due to its ability to integrate expert judgements and quantitative data into a transparent and systematic framework [6,15]. In the context of post-fire environmental assessment, AHP can be used to evaluate and prioritize criteria that influence ecosystem degradation. These criteria are often expressed in different units of measurement, requiring standardization to permit their comparison and facilitate mathematical operations for their evaluation and ranking. To accomplish this, the values are transformed to a uniform performance scale, which may be either discrete or continuous. Discrete scores represent the degree to which a specific factor influences the measured variable/outcome, typically categorized into predefined levels (e.g., low, medium, high). Continuous values, on the other hand, allow for more precise representation of a factors’ influence on a variable. These values are usually normalized (e.g., rescaled from 0 to 1) to ensure comparability across different units of measurement. Thresholds can be applied to continuous values to regulate their influence, maintain interpretability and ensure that factor contributions remain within a logical or empirically supported range. Scores and weights are then aggregated to construct the MCA model. An easy, yet efficient approach is the Simple Additive Weighting Model [1,16], which normalizes the criteria and sums the weighted scores for each model.

All models are sensitive to input parameters, either through uncertainty propagation or by changes in output due to small input variations [17]. Uncertainty analysis assesses how uncertainty in parameters affects results, while sensitivity analysis examines the extent to which variations in a parameter influence the outcome. Uncertainties in MCA arise mainly from subjective judgments in the selection and weighing of criteria. Therefore, sensitivity tests should be used to assess their robustness. While AHP’s CR metric ensures logical consistency in pairwise comparisons, it does not verify the stability of results under varying weights. Sensitivity analysis typically involves varying weights and scores to observe their impact on results, to determine the most influential parameters, and to identify changes that can affect outcomes. The simplest and widely applied type of sensitivity analysis requires testing one parameter at a time while keeping the other parameters constant (cf [9].). As an alternative to individual parameters’ assessments, random sampling (i.e., Monte Carlo simulation) can generate distributions around the weights, thereby evaluating the collective influence of all parameters on the model [17]. Besides sensitivity analysis, an important step is validating the model outputs against real data. While sensitivity analysis assesses the model’s robustness, field validation ensures the model meets specified performance standards under defined conditions, confirming its reliability. Note that field validation does not prove theoretical validity, and re-validation is necessary if the context changes [18].

### Study area and data definition

**Fig. 1:**
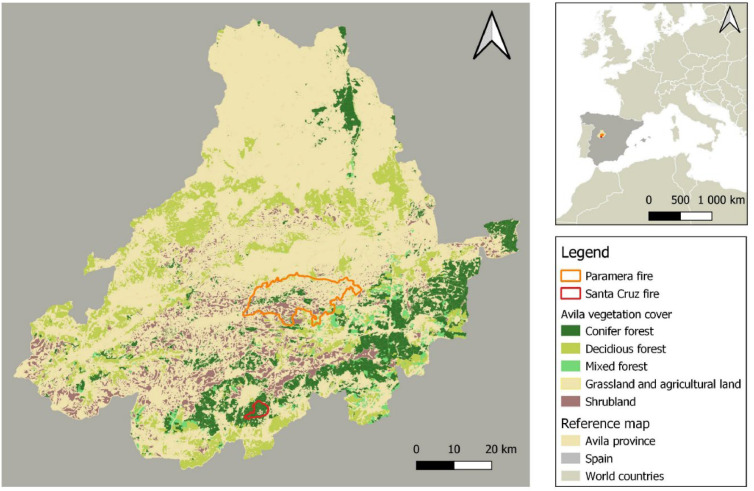
Main vegetation cover in the province of Ávila, and the two selected wildfire perimeters.

Post-fire environmental assessments are linked to geographical space, with geospatial data serving as a foundation for analysis. This work employs Multi-Criteria Analysis (MCA) to assess various ecological and environmental indicators derived from multiple data sources (Table 1), enabling the development of models for post-fire soil erosion risk and vegetation recovery potential. To ensure the applicability of these models, we validate them against field data from two wildfire events in the Ávila province, central Spain (Fig. 1).

**Table 1:** Data definition and sources.

Dataset (resolution)	Derived environmental variables (units)	Main source
**Digital Elevation Model (5m)**	**Slope (Slo, degrees):** steepness of the terrain affecting runoff speed, used in soil erosion risk evaluation.	Spanish Geographic Institute: https://centrodedescargas.cnig.es
	**Aspect (Asp, degrees):** Orientation of the slope that influences sunlight exposure and determines the suitability for plant growth and recovery.	
**Sentinel-2 images (10m spatial and 6-day temporal)**	**Fire severity index (FSI, unitless)**: bi-spectral index quantifying top vegetation layers’ changes after fire, indicating fire impact severity.	COPERNICUS repository: https://browser.dataspace.copernicus.eu
	**Bare soil index (BSI, unitless):** spectral index quantifying the reflectance of bare ground, indicating soil exposure after fire.	
**Global Aridity Index (30 arc-seconds)**	**Aridity levels (ordinal)**: climatic proxy reflecting precipitation and potential evapotranspiration, used to estimate suitability for plants’ regeneration.	[19]
**Burned area data (20m spatial and 6-day temporal)**	**Burned area (ha)**: polygon of the burned area.	European Forest Fire Information System (EFFIS): https://forest-fire.emergency.copernicus.eu
	**Historical fire impact (ordinal):** low, medium or high impact level, derived from the number of overlapping burned areas in a specific location in the last 20 years (frequency) and the time elapsed since the last fire (immaturity risk), used to estimate the ecosystem regeneration potential.	
Spanish Forest Map 2021 (MFE, 1:25,000)	**Post-fire regeneration strategy (RS, categorical)**: dominant species in each vegetation category (grass, shrub, and trees, excluding crops), classified based on their post-fire reproductive strategy (i.e., resprouters, post-fire seeders, seeders).	Ministry for Ecological Transition and the Demographic Challenge, Spanish Government www.miteco.gob.es/es/cartografia-y-sig/ide.html
	**Percentage of resprouting species (%Resp, numerical)** in each vegetation category used for post-fire erosion risk estimation.	
**Spanish Soil Erodibility Map 2011 (1: 50,000)**	**Soil erodibility factor K (Mg·h·ha^-1^·MJ^-1^·mm^-1^)**: Detachability and transportability of soil particles, used to estimate post-fire soil erosion risk.

### Factors influencing post-fire soil erosion

Post-fire soil erosion is typically triggered by the first rainfalls and is influenced by factors such as fire severity, exposed ground, soil erodibility, slope, and vegetation regrowth [20,21]. Given its episodic and context-specific nature, accurately predicting post-fire erosion using the (Revised) Universal Soil Loss Equation (USLE/RUSLE) is challenging [22]. Consequently, we engaged experts and stakeholders to identify the key factors influencing post-fire soil erosion in the study region, define their relationships to the erosion process through threshold setting, and assess their relative importance using an Analytic Hierarchy Process (AHP) weighting scheme.

#### Fire severity

Fire severity is defined as the degree to which a fire alters or disrupts an ecosystem, particularly through the consumption or loss of above- and belowground biomass, and its impacts on vegetation, soil structure, and organic layers [23,24]. Thus, it plays a critical role in post-fire soil erosion by determining primarily the extent of vegetation loss. The reduction of foliage cover increases the soil exposure to raindrop impact, while damage to plant roots reduces the soil’s binding capacity, heightening the risk of landslides as well as rill and gully erosion. Additionally, delayed vegetation recovery following severe fires by depleting seedbanks prolongs the period of erosion risk. The Composite Burn Index (CBI), relying on expert estimations, is a widely employed field-based measure of burn severity. However, field assessments can be challenging, particularly for large fire-affected areas or inaccessible terrain. To address these challenges, several remote sensing indices have been developed to estimate burn severity from satellite imagery captured before and after fire events. Among these, the differenced Normalized Burn Ratio (ΔNBR) is particularly prominent due to its strong correlation with CBI, making it highly effective for use across large spatial scales [25,26]. However, the strength of these correlations can vary depending on vegetation type, fire severity, and landscape characteristics.

We relied on the Copernicus repository and Google Earth Engine (GEE) to compute the ΔNBR based on Sentinel-2 pre- and post-fire satellite imagery:

$$\Delta NBR=\;NBR_{pre\_fire}-NBR_{post-fire}\;\text{Where}:\;NBR=\;\left(NIR-SWIR\right)/\left(NIR+SWIR\right)$$

*with NBR denoting the Normalized Burn Ratio, NIR the near-infrared band, and SWIR the shortwave infrared band.*

**Fig. 2:**
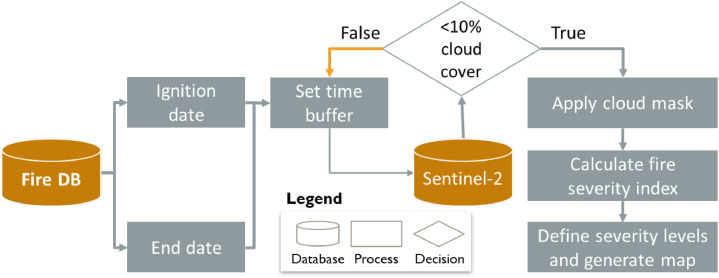
Fire severity calculation workflow: 1. Connect to the fire database and select fire dates. 2. Set an initial time buffer before and after fire to search for satellite images with <10 % cloud cover; if condition is not satisfied, increase the time buffer. 3. Apply cloud mask if needed, 4. Calculate fire severity index ΔNBR or ΔNDVI based on selected pre- and post-fire satellite images, and 5. Define severity levels and generate fire severity map.

Burned area information was obtained from the EFFIS wildfire dataset [27], where fire ignition and end dates were used, with an initial time buffer of 30 days, to select the images. Images with >10 % cloud cover were excluded. A cloud mask was applied, and a cloud-free mosaic was generated before computing the ΔNBR. Fire severity levels were then classified based on the thresholds defined by [25], with values <0.1 corresponding to unburned areas, while greater that 0.66 to high severity burns (Fig. 2).

#### Bare soil index

High-severity fires remove vegetation cover and organic litter, leaving the soil exposed and vulnerable to raindrop impact. Additionally, severe fires can alter soil properties by disrupting soil aggregates and increasing water repellency (hydrophobicity), which reduces water infiltration, enhances surface runoff, and accelerates erosion processes such as sheet, rill, and gully erosion [28]. Since spectral Fire Severity Indices (FSI) primarily assess vegetation cover, we incorporated the Bare Soil Index (BSI), which enhances the contrast between soil and vegetation, to better account for soil exposure:

BSI=(SWIR+RED)+(NIR+BLUE)/(SWIR+RED)−(NIR+BLUE)

*where: SWIR is the shortwave Infrared band; RED is red band; NIR is the near-Infrared band and BLUE is the blue band.*

#### Vegetation regrowth

Vegetation regrowth in the first months following a fire helps mitigate erosion by stabilizing the soil through root systems and reducing surface runoff [29]. Resprouting species are typically the first to regenerate, as they can rapidly resprout from protected buds in roots or stems, thereby promoting soil stabilization soon after a fire event. As a result, the percentage of resprouting species can be used as a reliable proxy for the early stages of vegetation regrowth [24,30]. The post-fire strategies of plant species were identified using plant trait databases, along with a comprehensive review of peer-reviewed literature (see Regeneration Capacity section). We estimated the percentage of resprouting species based on the most recent Spanish Forest Map [31], that provides species distribution data for each homogeneous vegetation stratus (MFE polygon). First, we recorded the number of resprouting species in each MFE polygon then multiplied by their spatial coverage and divided by the polygon’s total area. We established the variable’s threshold based on [12,32]: if at least 40 % of a map polygon is covered by resprouting species, its contribution to erosion is considered minimal, as these species help mitigate erosion within a few months after a wildfire.

#### Soil erodibility

The soil erodibility factor (K) reflects both soil detachability and runoff potential and is a key component in the Universal Soil Loss Equation (USLE) and its revised version, RUSLE (Revised Universal Soil Loss Equation). Soils rich in clay have low infiltration rate, but are resistant to detachment, while soils rich in sand have high infiltration rate but are easily detached. Consequently, both types of soils tend to have relatively low K values. Silt is generally considered the most erodible particle, as it is both reasonable to detach and carry [33]. Empirical studies indicate that wildfires can significantly increase soil erodibility in forested areas [5,34]. Therefore, we set the upper limit of erodibility within burned areas to 0.55 Mg h ha^-1^ MJ^-1^mm^-1^, which corresponds to the maximum value observed in the Ávila province, including agricultural land.

#### Slope

Water erosion occurs mostly on slopes, where raindrops weaken soil cohesiveness and release particles, which are subsequently carried downslope by surface runoff. The severity of erosion is thus determined by rainfall intensity, soil texture, vegetation cover, slope angle, and length. Steeper slopes facilitate faster water flow, increasing erosive force, while longer slopes allow water to accumulate, increasing runoff volume. We calculated slope using the 5 m Digital Elevation Model (DEM). Based on expert knowledge and regional context, we consider slopes between 0 % and 9 % as contributing minimally to soil erosion, whereas slopes of 80 % or steeper representing the upper threshold for erosion susceptibility [35].

### Factors influencing post-fire vegetation recovery

In a post-fire landscape, the ecosystem recovery depends on pre-fire vegetation modulated by the fire damage and the post-fire environmental factors [2]. We considered the species-specific regeneration capacity, which determine the inherent ability of vegetation to resprout or recruit from seed; fire severity, which quantifies the extent of damage sustained by vegetation; climatic conditions, represented by the aridity index, which indicates water availability for regrowth; slope orientation (aspect), which affects microclimatic conditions that regulate plant establishment; and fire history, as it can determine vegetation maturity and soil degradation from previous fire events.

#### Fire severity

In the context of post-fire vegetation recovery, fire severity refers to the degree of vegetation damage, but can also encompass changes in soil properties, and the alteration of ecosystem processes. While field assessments can address all these aspects, remote sensing methods (e.g., the ΔNBR index) primarily focus on changes in canopy cover after fire [25]. Low-severity fires typically burn surface litter and understory vegetation while preserving tree canopies, allowing for rapid recovery through resprouting and seed germination. In contrast, high-severity fires cause complete canopy loss and can harm soil seed banks, leading to delayed or failed forest regeneration, especially in dry climates where seed sources are scarce (Table 2). Studies in Mediterranean forests [36,37] have shown that high-severity burns can shift forests toward shrub-dominated landscapes, that may also increase fire recurrence risk. Additionally, severe fires can cause soil erosion, hydrophobic conditions, and loss of essential nutrients, further hindering vegetation recovery in some ecosystems [38].

**Table 2:** Fire severity levels based on Key and Benson [25] and impact on vegetation recovery.

Fire severity level	ΔNBR range	CBI range	Vegetation impact
**Unburned**	< 0.1	0	No impact: no significant vegetation damage
**Low**	0.1 – 0.27	0.1 – 1.24	Low impact: light surface burns, minimal canopy loss
**Moderate**	0.27 – 0.66	1.25 – 2.24	Moderate impact: partial canopy damage, understory affected
**High**	> 0.66	2.25 – 3.0	High impact: complete canopy loss, high tree mortality

#### Regeneration capacity

**Fig. 3:**
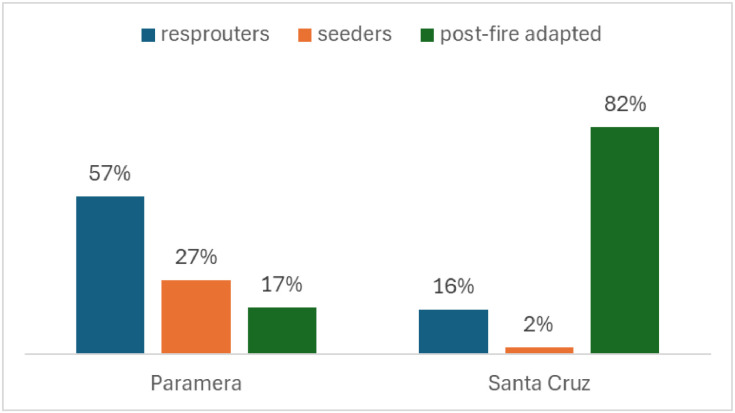
Percentage of species-specific post-fire regeneration categories in the two burned areas.

In this study, we define regeneration capacity per land unit as the cumulative vegetation regeneration, determined by species-specific fire response strategies. We identified fire response strategies for plant species and species groups listed in the Ávila province Forest Map (MFE, 2017), based on plant trait databases BROT [39] and FEIS [40], and supplemented by peer-reviewed literature. For species lacking fire adaptation data, we used their reproductive strategy as a proxy. Fig. 3 illustrates the distribution of fire response strategies within the two selected wildfires (see Fig. 1). Species functional groups were then ranked based on their regeneration potential: (i) resprouters, which regenerate from roots or surviving structures after fire; (ii) post-fire seeders, which rely on fire-stimulated seed germination; and (iii) seeders, which depend on seed dispersal from unburned patches or nearby areas (Table 7). Finally, we estimated the regeneration capacity by integrating vegetation categories and regeneration potential within MFE polygons, applying a weighted sum, with weights assigned according to the proportional cover of each species within the mapped polygon:

$$RC=\;\frac{\sum_{i}^{3}\left(DT_{i}\times C_{i}\right)+\;S\times C_{S}\;+\;G\times C_{G}}{\sum_{i}^{3}C_{i}\;+\;C_{S}\;+\;C_{G}}$$

*Where RC is the regeneration capacity; DT is the scored regeneration potential for the dominant tree species; Ci is the occupancy of each tree species in the map polygon (MFE provides 3 co-dominant tree species per stratum); S and G are the regeneration scores for the shrub and grass layers respectively, CS is the shrub cover; and CG is the grass cover*.

#### Aridity index

Aridity levels determine the hydrological response of burned landscapes, with higher aridity associated with reduced post-fire infiltration, increased surface runoff, and elevated desertification risks [41,42]. Higher levels of aridity (i.e., reduced moisture availability) in post-fire landscapes could lead to a shift in vegetation from forests to shrublands [36]. Moreover, in arid environments, delayed vegetation recovery prolongs soil exposure, increasing erosion risks and hindering plant regeneration, which reinforces a negative feedback loop that further slows ecosystem recovery [43,44]. In this study we used the Global Aridity Index (GAI [19],) and adjusted the aridity levels based on the UNEP (United Nations Environment Programme) classification, to reflect the climate of our study area. These adjustments and the relevance of the GAI to vegetation recovery are supported by the main references, as detailed in Table 3. GAI estimates the precipitation deficit relative to atmospheric water demand in a given area. It quantifies how much water is available in an ecosystem compared to the amount needed by plants for transpiration and other processes, acting as a proxy for water availability and climatic stress on vegetation. Lower GAI values correspond to drier conditions, where limited rainfall and high evapotranspiration rates restrict vegetation growth, while higher GAI values indicate more stable precipitation patterns and denser vegetation cover.

**Table 3:** Aridity levels and their impact on post-fire vegetation recovery.

Aridity level	Impact on vegetation recovery	Explanation	Main references
**Arid or Hyper-arid** **(AI < 0.21)**	High: very slow or absent recovery	• Extremely low water availability limits plant regrowth. • Fire-adapted species may struggle to re-establish. • High evaporation rates cause soil degradation and desertification risks.	D’Odorico et al. [45,46]
**Semi-arid** **(AI = 0.21– 0.5)**	Moderate: partial, slow recovery	• Water availability fluctuates, leading to uneven plant regeneration. • Fire-adapted species regrow, but tree recovery can be slow. • Repeated fires can lead to shrub encroachment or grassland dominance	Baudena et al. [36]
**Low** **(AI > 0.5)**	Low: Full, fast recovery	• Higher precipitation supports rapid regrowth of trees and understory plants. • Increased soil moisture promotes seed germination and root recovery	Pausas et al. [47]

#### Aspect

Aspect defines microclimatic conditions such as solar radiation, temperature, and evapotranspiration, which in turn regulate vegetation recovery and soil moisture availability. In warm climates in the Northern Hemisphere, south-facing slopes receive higher solar radiation, leading to increased evapotranspiration, reduced soil moisture, and slower vegetation regrowth. In contrast, north-facing slopes receive less direct sunlight, resulting in lower evapotranspiration and higher soil moisture retention, supporting faster vegetation recovery. East- and west-facing slopes experience intermediate conditions, with vegetation recovery potential varying depending on regional climate and seasonal precipitation patterns. To incorporate these factors, we classified aspect into three categories, as shown in Table 4.

**Table 4:** Aspect categories and their impact on vegetation recovery after fire.

Aspect	Impact on vegetation recovery	Explanation	Main references
**N, NE, NW**	Low (score 1): Full, fast recovery	Less solar radiation is more favorable for plant development since more water is retained through transpiration effects.	Alloza et al. [12]; Duguy et al. [10]; Underwood et al. [11]
**SW, SE**	Moderate (score 2): partial, slow recovery	• Sun exposure on SE, SW slopes cause lower evapotranspiration than south-facing slopes, moderately retaining moisture.	
**S**	High (score 3): very slow recovery	• Higher solar radiation increases evapotranspiration, leading to drier soils. • Lower soil moisture retention inhibits seed germination and plant regrowth.	

#### Fire history

The interplay between fire frequency and the time elapsed since the last fire is key for assessing ecosystem resilience and vegetation recovery. Fire frequency influences ecosystem composition, nutrient cycling, and vegetation recovery, and different plant species have evolved to tolerate or even depend on specific fire intervals [48,49]. However, fires occurring at short intervals can hinder vegetation regeneration by heightening the immaturity risk, i.e., the likelihood that a plant community will experience recurrent fire before it has fully recovered and reached reproductive maturity [50]. Conversely, longer periods without fire allow ecosystems to recover. Following Alloza et al. [12], we categorize the fire history impact of vegetation recovery potential based on fire frequency, i.e., the number of overlapping burned areas at a given location over the past 20 years, and time elapsed since the last fire, which indicates immaturity risk, as shown in Table 5.

**Table 5:** Fire history score table, adapted from Alloza et al. [12].

The Low category was assigned a score of 1, the Medium category a score of 2, and the High categories a score of 3, ensuring consistency with the scale applied to other variables in the vulnerability assessment analysis.

### Multi-criteria post-fire environmental evaluation

We employed the Analytical Hierarchy Process (AHP) to derive relative weights for factors affecting post-fire soil erosion and vegetation recovery. Three academic experts and two stakeholder representatives (Suplementary Table 3) conducted pairwise comparisons, assigning relative importance scores to five factors of soil erosion and five factors of vegetation recovery, ensuring a CR < 0.1 (Supplementary Table 4).

Using the defined thresholds (Table 6), we normalized criteria influencing post-fire soil erosion risk to a standardized scale 0 to 1. We then assigned discrete scores to the factors influencing post-fire vegetation recovery (Table 7).

**Table 6:** Thresholds for factors influencing soil erosion risk based on literature or expert knowledge.

Post-fire soil erosion risk					
*Criteria*	*Fire severity index (unitless)*	*Erodibility factor K (Mg h ha^-1^* MJ*^-1^mm^-1^)*	*Bare Soil Index (BSI, unitless)*	*Slope (%)*	*Resprouters (% cover)*
*Threshold*					
Min	0.1	0	−1	9	0
Max	0.66	0.55	1	80	0.4

**Table 7:** Discrete scores for criteria influencing post-fire vegetation recovery.

Post-fire vegetation recovery						
*Criteria*	*Fire severity index*	*Aspect*	*Aridity Index*	*Fire history*		*Regeneration strategy*
*Score*				*Fire frequency*	*Last fire interval*	
1	0.1 – 0.26	N, NE, NW	> 0.5	0 - 1	> 15	Resprouters
2	0.27 – 0.65	SE, SW	0.5 – 0.2	1 - 2	10 - 15	Fire-adapted seeders
3	> 0.66	S	< 0.21	> 2	< 10	Seeders

**Fig. 4:**
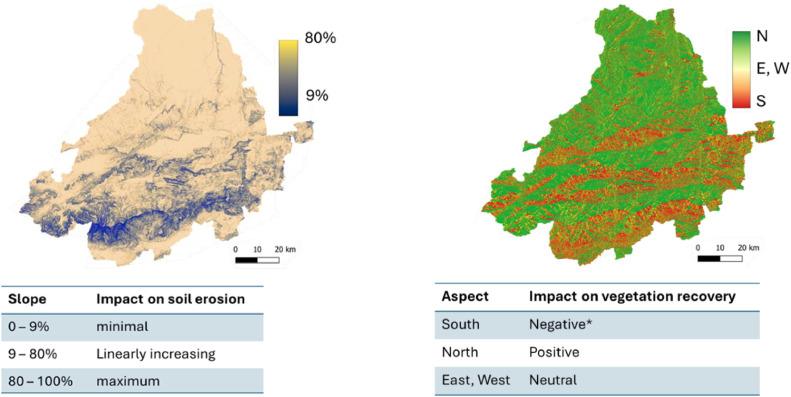
Applying thresholds (left) and scores (right) to slope and aspect raster files respectively.

Fig. 4 illustrates how thresholds and scores were applied to georeferenced raster images representing DTM-derived factors. For instance, slope (Fig. 4, left) was scaled between 9 % and 80 %, assuming minimal impact on soil erosion risk below 9 %, maximum impact above 80 %, and a linear increase in impact between these values. Similarly, aspect was evaluated based on its influence on vegetation recovery potential. South-facing slopes were assigned a score of 3, as increased sun exposure and higher evapotranspiration in the study area create less favorable conditions for plant growth. In contrast, north-facing slopes retain more moisture, fostering better vegetation recovery, and were therefore assigned a score of 1.

Finally, the factor scores, combined with the expert-derived weights were applied in the simple additive weighting model [1,16] to obtain the composite index of post-fire soil erosion risk:

$$SER=FSI\times w_{FS}+K\times w_{k}+BSI\times w_{BSI}-PR\times w_{PR}+Slo\times w_{Slo}$$

*where SER is the post-fire soil erosion risk, K is the erodibility factor, FSI the fire severity index, BSI is the bare soil index, PR is the percent or resprouting species, Slo the slope, w_FSI_, w_BSI_, w_PR_ and w_Slo_ their respective weights*. and post-fire vegetation recovery potential:

$$VRP=RC\times w_{RC}+FSI\times w_{FSI}+AI\times w_{AI}+Asp\times w_{Asp}+FH\times w_{FH}$$

*where VRP is the post-fire vegetation recovery potential, RC is the species-specific post-fire regeneration capacity, FSI is the fires severity index, AI the aridity index, Asp the aspect, FH the fire history, w_RC_, w_FSI_, w_AI_ w_Asp_ and w_FH_ the respective weights*.

## Method validation

### Field assessment

**Fig. 5:**
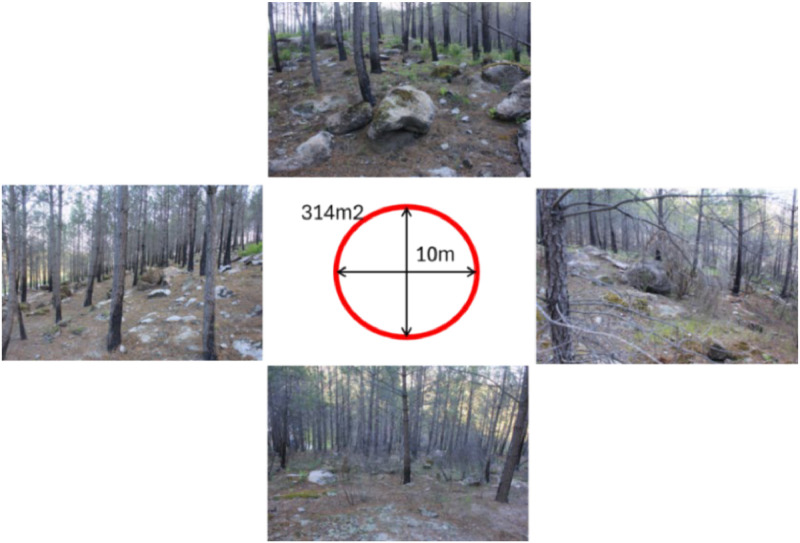
Plot representation: From each plot centre, we measured 10 m in four directions (downslope, upslope, right, left) using a measuring tape and took photographs following the inclination of the terrain.

The MCA framework was validated against field data collected in April 2024 from two wildfire-affected areas in the province of Avila, central Spain (Fig. 1). The first wildfire occurred in August 2021 in the Sierra de la Paramera, burning over 22.000 hectares of shrubland and grassland. The second fire ignited in August 2022 near Santa Cruz del Valle, affecting 1.500 hectares of *Pinus pinaster* forest. Field measurements to collect vegetation recovery data and soil erosion observations were conducted in 11 sites in Paramera and 13 sites in Santa Cruz, with each site defined as a 10-m radius circular plot (Fig. 5). Vegetation cover was estimated based on a reference chart [51], and observed soil erosion was recorded based on erosion types described in Table 8. Human or animal impacts within these sites were categorized into soil compaction (i.e., machinery, logging), overgrazing, or no observable impact (Table 8). Observed erosion types were scored from 0 to 1 based on erosion intensity (Table 8), and the highest value per plot was retained. Vegetation recovery was quantified by combining shrub and grass cover estimations, weighted by their average heights: 120 cm for shrub, 10 cm for grass strata. Observations on standing trees were omitted to focus on early-successional vegetation, detectable 2–3 years after a fire.

**Table 8:** Erosion types and their intensity score.

Erosion Categories	Description	Score
No erosion (NE)	No signs of any type of erosion are observed	0
Weak diffuse erosion (EDD)	Loss of fine material on the surface due to sheet runoff	0.2
Strong diffuse erosion (EDF)	Sheet erosion with greater impact. Areas without fine material are observed	0.4
Wind erosion (EO)	Removal of soil particles by wind, often leaving a compacted or deflated surface	0.5
Weak rill erosion (XDD)	Small rills or channels caused by water flow, shallow in depth.	0.6
Strong rill erosion (XDF)	Well-defined and deeper rills (channels) due to faster water flow	0.8
Collapses (ESF)	Large-scale soil detachment and mass movement, often forming gullies or landslides	0.9

### Validation and sensitivity analysis

Measured soil erosion and vegetation cover were compared with MCA-derived values of SER and VRP, estimated immediately after the fire event. For each plot, center coordinates with a 10-meter buffer were used to sample maps and extract average values, enabling comparison between observed and predicted data. The dataset was assessed for compliance with linear modeling assumptions, and statistical analyses were conducted: Spearman’s correlation to evaluate rank-based associations, ANOVA to compare group differences by locality and human/animal impact, and linear regression to quantify the relationship between observed and MCA-predicted values. We then compared the maps generated using the weighting schemes of individual AHP participants and those derived from the average weights (Supplementary Table 4). The use of average weights was assumed to provide a more balanced representation of participant judgments, reflecting a democratic aggregation of individual perspectives. Finally, we applied Monte Carlo simulation with random sampling to introduce controlled variability in the weights of each model parameter by ±10 %, ±20 %, and ±30 % (cf [17].). By analyzing the distribution graphs, we identified the sensitivity thresholds of the models to parameter weight variations within ±20 %. Beyond this range, larger fluctuations resulted in shifts in relative priorities, thereby compromising the stability of the models. All analyses were performed in R Version 4.4.2 [52], with ggplot2 [53] for visualization and lme4 [54] for linear modelling.

**Fig. 6:**
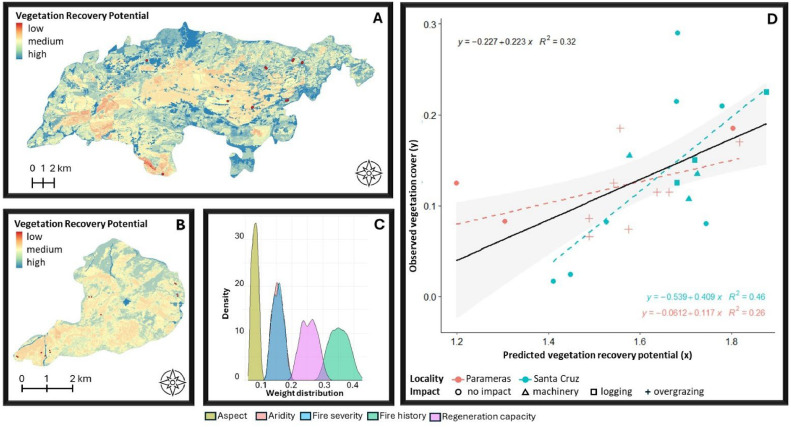
Vegetation Recovery Potential Scenario A – Best scenario for vegetation recovery (A) in Paramera and (B) in Santa Cruz, exhibiting the highest R-squared value (D) and relatively distinguishable weight distributions with a random 20 % variation around the weights (C) - indicating stability in response to small variations (±20 %). The resulting maps (A and B) show moderate to high vegetation recovery potential and the red dots illustrate the sample plots.

**Fig. 7:**
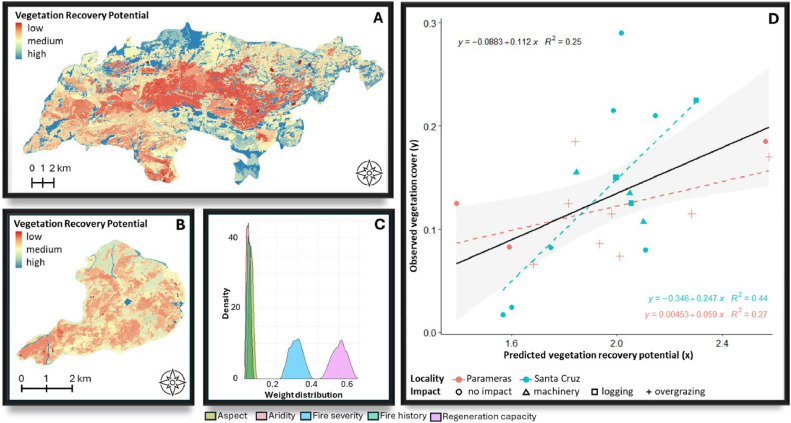
Vegetation Recovery Potential Scenario B – Worst scenario for vegetation recovery (A) in Paramera and (B) in Santa Cruz, exhibiting the lowest R-squared value (D) and overlapping weight distributions due to random 20 % variation in aspect, aridity, and fire history, suggesting interchangeable factor priorities. In contrast, fire severity and regeneration capacity show distinguishable distributions, indicating stability within 20 % weight variation (C). The resulting maps (A and B) display low to high vegetation recovery potential, with red dots marking the sample plots.

**Fig. 8:**
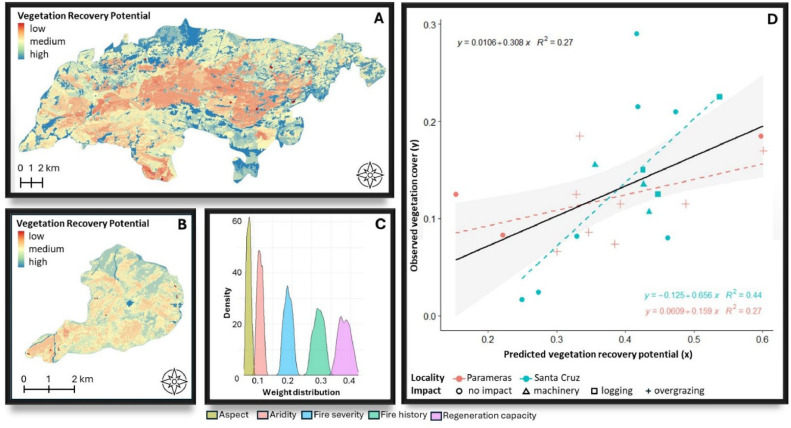
Vegetation Recovery Potential Scenario C - the "democratic scenario," averages the weights from all AHP participants, resulting in distinguishable distributions (C) that indicate stability in parameter input variation (±20 %). The resulting maps (A and B) display moderate to high vegetation recovery potential.

**Fig. 9:**
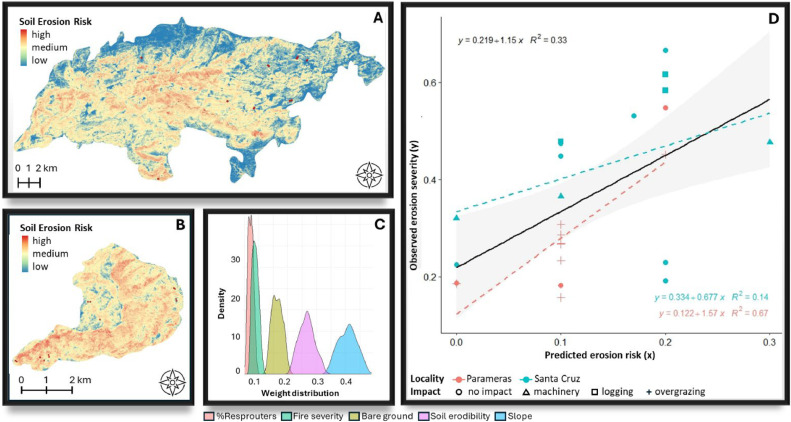
Soil erosion risk scenario A: Best scenario for soil erosion risk in (A) Paramera and (B) Santa Cruz, with maps displaying soil erosion risk levels (low to high), and red dots marking sample plots. This scenario represents the highest R² value (D). Weight distributions remain relatively distinct with 20 % randomization, except for overlapping %resprouters and fire severity, which have minimal model impact (C).

**Fig. 10:**
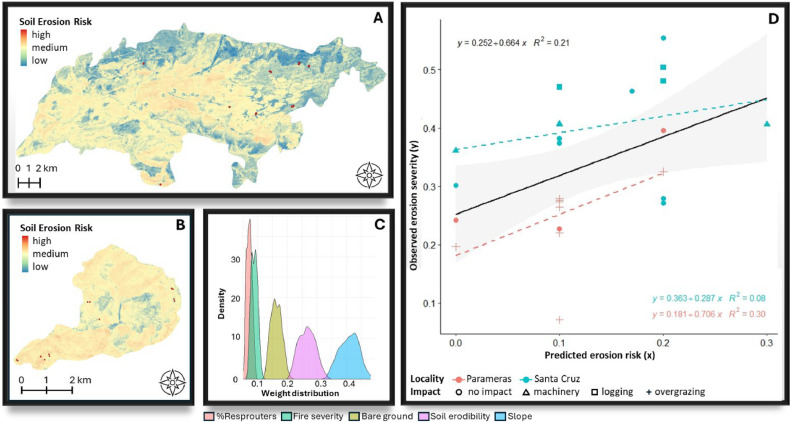
Soil erosion risk scenario B: Worst scenario for soil erosion risk i in (A) Paramera and (B) Santa Cruz, with maps displaying soil erosion risk levels (low to high), and red dots marking sample plots. This scenario represents the lowest R² value (D). Weight distributions remain relatively distinct with 20 % randomization, except for overlapping %resprouters and fire severity, which have minimal model impact (C).

**Fig. 11:**
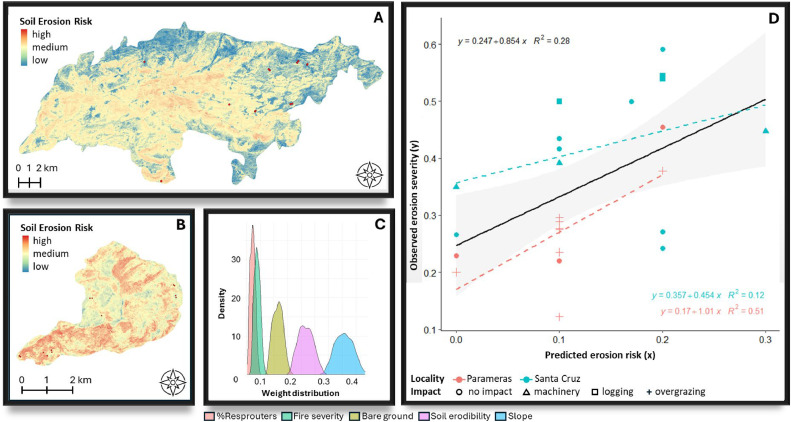
Soil erosion risk scenario C: the ‘democratic’ scenario for soil erosion risk i in (A) Paramera and (B) Santa Cruz, with maps displaying soil erosion risk levels (low to high), and red dots marking sample plots. This scenario represents the averaged weights, and field validation resulted in (D). Weight distributions remain relatively distinct with 20 % randomization, except for overlapping %resprouters and fire severity, which have minimal model impact (C).

Fig. 6, Fig. 7, Fig. 8, Fig. 9, Fig. 10, Fig. 11 illustrate the validation framework through scenario comparison. A total of six models, corresponding to each weighting scheme as well as the average weights model, were validated against field data. The Spearman correlation analysis revealed a positive correlation in all cases (mean Spearman’s rho = 0.52, SD = 0.05). Although the linear regression visualizations (Fig. 6, Fig. 7, Fig. 8, Fig. 9, Fig. 10, Fig. 11D) indicated variations in regression lines based on locality, ANOVA analysis showed no significant influence of locality or management practices (p-value > 0.1 in all cases). Fig. 6, Fig. 7, Fig. 8, Fig. 9, Fig. 10, Fig. 11 illustrate the best-fit model (Scenario A), the worst-fit model (Scenario B), and the averaged weights model (Scenario C) for both vegetation recovery potential (Fig. 6, Fig. 7, Fig. 8) and soil erosion risk (Fig. 9, Fig. 10, Fig. 11).

## Limitations

Participatory Multi-Criteria Analysis (MCA) is inherently subjective, as demonstrated by the variability in experts’ rankings of environmental factors. This lack of consensus reflects a fundamental challenge in participatory decision-making processes, where expert judgments are shaped by disciplinary backgrounds, professional experience, and cognitive biases [15,55]. Integrating diverse perspectives into environmental evaluations contributes to uncertainty in the possible outcome. Furthermore, methodological assumptions amplify these uncertainties by imposing predefined frameworks that may not fully capture the complexity of environmental systems. The Simple Additive Weighting Method (SAWM), commonly used in MCA, operates under the assumption of linearity and additivity, treating individual criteria/factors as independent contributors to the final evaluation [7]. Yet, natural ecosystems often exhibit non-linear interactions and interdependencies, which are challenging to capture within this framework. Despite its widespread use in direct environmental impact assessments, MCA may be effective as a tool for scenario testing [15]. Another critical challenge lies in the data limitation. For instance, the vegetation recovery potential assessment relies on vegetation data from the Spanish Forest Map (MFE), derived from national forest inventories and photointerpretation, which introduce uncertainties at fine spatial scales, and omit understory vegetation—an essential factor in post-fire recovery that remains unaccounted for . Similarly, spectral fire severity indices mainly reflect changes in upper vegetation layers, leading to over- or under-estimation of fire impacts [[56], [57], [58]]. On the other hand, the validation of the method was conducted using a total of 26 plots across two wildfire-affected areas, which, while comparatively limited for a comprehensive statistical analysis, was constrained by inaccessibility and time limitations. Although this may impact the interpretability of the results, it does not compromise the primary objective of validation: demonstrating that MCA-based outputs are meaningfully related with observed vegetation recovery and erosion risk. The above limitations present an opportunity for the development of data-driven approaches to enhance the accuracy of post-fire environmental assessments by systematically quantifying the influence of environmental and site-specific factors.

## Ethics statements

Nothing to state

## CRediT author statement

**Irina Cristal**: Methodology, Conceptualization, Software, Validity test, Visualizations, Writing- Original draft preparation. **Elena Puigdemasa**: Conceptualization, Methodology, Field Assessment, Data curation, Writing- Reviewing and Editing. **Marina Palmero-Iniesta**: Conceptualization, Methodology. **Pere Pons**: Conceptualization, Methodology, Field Assessment, Writing- Reviewing and Editing, Funding Acquisition.

## Declaration of competing interest

The authors declare that they have no known competing financial interests or personal relationships that could have appeared to influence the work reported in this paper.

## Data Availability

The data and code for this research are available in public repository DOI: 10.5281/zenodo.15119754.

## Appendix Supplementary materials

Image, application 1
