# Supplementary material for: Post-fire environmental assessment: a participatory multi-criteria approach for estimating soil erosion risk and vegetation recovery potential
Source: MethodsX. 2025 Sep 23;15:103647. doi: 10.1016/j.mex.2025.103647 (PMC12538923; doi:10.1016/j.mex.2025.103647)
Supplement: Supplementary file 1 [file mmc1.docx]

# Supplementary material and additional information

The present study provides supporting methods, data, and additional analysis for the following co-submitted article:

*I. Cristal, E. Puigdemasa, M. Palmero-Iniesta, E. Mauri, P. Pons, Enhancing Post-Fire Decision-Making: A Framework for Rapid Wildfire Impact Assessment and Evidence-Based Management Planning. Available at SSRN:*[*https://ssrn.com/abstract=5191996*](https://ssrn.com/abstract=5191996)*or*[*http://dx.doi.org/10.2139/ssrn.5191996*](https://dx.doi.org/10.2139/ssrn.5191996)

Supplementary table 1. Saaty's comparison scale

| Value | Interpretation |
| --- | --- |
| 1 | Equal importance |
| 3 | Moderate importance |
| 5 | Strong importance |
| 7 | Very strong importance |
| 9 | Extreme importance |
| 2,4,6,8 | Intermediate values |

Note: if the criterion in the row is significantly more important than the criterion in the column, then $a_{ij}=5$, and consequently, $a_{ji}$ will automatically be $\frac{1}{5}$

Supplementary table 2. Random Index reference values based on the number of criteria n

| ***n:*** | 1 | 2 | 3 | 4 | 5 | 6 | 7 | 8 | 9 | 10 |
| --- | --- | --- | --- | --- | --- | --- | --- | --- | --- | --- |
| ***RI:*** | 0 | 0 | 0.58 | 0.90 | 1.12 | 1.24 | 1.32 | 1.41 | 1.45 | 1.49 |

Supplementary table 3. Experts' profiles and their contribution in the multi-criteria evaluation.

| **Expert profile** | **Contribution** |
| --- | --- |
| 1. **Ecology** | Knowledge of ecological principles, focus on biodiversity, habitat conservation, and ecosystem functions. |
| 1. **Forest Management** | Knowledge of Mediterranean forests, focus on wildfire risk, resilience, and sustainable management practices. |
| 1. **Stakeholder Group Representation** | Represented the priorities and perspectives of local stakeholders from the study area (Avila), ensuring alignment with community-specific concerns. |
| 1. **Opinion Group Representation** | Voiced the consensus of a multidisciplinary expert group that independently ranked and weighted criteria outside the AHP framework. |
| 1. **Environmental Sciences** | Specialized in forest ecology and spatial analysis. |

*Supplementary table 4. Criteria, weights and the consistency ratio derived from the five participants.*

| **Factors** | | **Expert 1** | **Expert 2** | **Expert 3** | **Expert 4** | **Expert 5** | **Average** |  |
| --- | --- | --- | --- | --- | --- | --- | --- | --- |
|  | **Soil erosion risk** | | | | | | | |
| Slope | | 0.423 | 0.523 | 0.291 | 0.307 | 0.408 | 0.390 |  |
| Soil erodibility (K factor) | | 0.173 | 0.13 | 0.462 | 0.455 | 0.267 | 0.297 |  |
| Bare ground (BSI) | | 0.27 | 0.048 | 0.098 | 0.041 | 0.164 | 0.124 |  |
| Fire severity (ΔBNR) | | 0.102 | 0.225 | 0.04 | 0.061 | 0.089 | 0.103 |  |
| % Resprouters | | 0.033 | 0.075 | 0.109 | 0.137 | 0.072 | 0.085 |  |
| Consistency ratio (CR) | | 0.03 | 0.09 | 0.08 | 0.04 | 0.09 | 0.07 |  |
|  | **Vegetation recovery potential** | | | | | | | |
| Regeneration strategy | | 0.256 | 0.506 | 0.426 | 0.452 | 0.036 | 0.335 |  |
| Fire recurrence | | 0.355 | 0.065 | 0.078 | 0.361 | 0.482 | 0.268 |  |
| Fire Severity | | 0.154 | 0.294 | 0.309 | 0.048 | 0.143 | 0.190 |  |
| Aridity | | 0.154 | 0.06 | 0.077 | 0.062 | 0.237 | 0.118 |  |
| Aspect | | 0.081 | 0.075 | 0.111 | 0.076 | 0.102 | 0.089 |  |
| Consistency ratio (CR) | | 0.01 | 0.05 | 0.08 | 0.04 | 0.09 | 0.06 |  |

Supplementary table 5. Field data

| **Locality** | **X** | **Y** | **Erosion type** | **%Shrub** | **%Grass** | **Impact** |
| --- | --- | --- | --- | --- | --- | --- |
| Santa Cruz | 328869.88 | 4453024.93 | NE, EDD, EDF | 15 | 30 | Machinery tracks/soil Compaction |
|  | 328827.16 | 4453058.23 | NE, EDD, EDF | 5 | 20 |  |
|  | 330067.09 | 4453201.31 | NE, XD | 5 | 75 |  |
|  | 330051.47 | 4453273.30 | EDD, NE, EDF | 10 | 30 | Log skidding |
|  | 329887.43 | 4453067.97 | NE, EDD | 10 | 5 |  |
|  | 329738.42 | 4453187.28 | EDF, EDD, NE | 15 | 45 |  |
|  | 331511.18 | 4455250.00 | NE | 1 | 95 | Machinery tracks/soil, Compaction |
|  | 331599.53 | 4455248.03 | NE, EDD | 10 | 35 |  |
|  | 331956.97 | 4454587.47 | NE | 1 | 70 |  |
|  | 334779.14 | 4455353.53 | EDD, EDF, NE | 1 | 5 |  |
|  | 334793.32 | 4455283.47 | EDD, NE, EDF | 2 | 0.5 |  |
|  | 334737.95 | 4455726.26 | NE, EDD | 15 | 35 |  |
|  | 334694.00 | 4455758.54 | NE, EDD | 20 | 50 |  |
| Parameras | 353193.23 | 4487741.16 | NE, EDD | 1 | 54 | Machinery tracks/soil, Compaction |
|  | 353215.76 | 4487796.47 | NE, EDD | 10 | 65 |  |
|  | 349968.80 | 4487262.10 | NE, EDD | 2 | 50 |  |
|  | 349874.81 | 4487338.73 | NE | 0.5 | 80 |  |
|  | 352334.19 | 4487926.46 | EDD, NE | 5 | 65 |  |
|  | 352414.94 | 4488058.79 | EDD, NE | 5 | 55 | Overgrazing |
|  | 351900.69 | 4484379.57 | NE, EDD | 3 | 47 |  |
|  | 351739.77 | 4484336.93 | NE | 5 | 65 |  |
|  | 348678.22 | 4483691.99 | NE, EDD, EDF | 10 | 65 |  |
|  | 348722.54 | 4483759.39 | NE, EDD, EDF | 10 | 50 |  |
|  | 346444.11 | 4484294.60 | NE, EDD | 5 | 55 |  |
